# Vision-language models for automated video analysis and documentation in laparoscopic surgery: a proof-of-concept study

**DOI:** 10.1097/JS9.0000000000003069

**Published:** 2025-07-17

**Authors:** Esther Helene Stueker, Fiona R. Kolbinger, Oliver Lester Saldanha, David Digomann, Steffen Pistorius, Florian Oehme, Marko Van Treeck, Dyke Ferber, Chiara Maria Lavinia Löffler, Jürgen Weitz, Marius Distler, Jakob Nikolas Kather, Hannah Sophie Muti

**Affiliations:** a Else Kröner Fresenius Center for Digital Health, Dresden University of Technology, Dresden, Germany; b Department for Visceral, Thoracic and Vascular Surgery, University Hospital and Faculty of Medicine Carl Gustav Carus, Technische Universität Dresden, Dresden, Germany; c Weldon School of Biomedical Engineering, Purdue University, West Lafayette, USA; d Department of Biostatistics and Health Data Science, Richard M. Fairbanks School of Public Health, Indiana University, Indianapolis, USA; e National Center for Tumor Diseases Dresden (NCT/UCC), a partnership between DKFZ, Faculty of Medicine and University Hospital Carl Gustav Carus, TU Dresden University of Technology and Helmholtz-Zentrum Dresden - Rossendorf (HZDR), Dresden, Germany; f German Cancer Consortium (DKTK), Partner Site Dresden, German Cancer Research Center (DKFZ), Heidelberg, Germany; g Asklepios-ASB Krankenhaus Radeberg, Radeberg, Germany; h Department of Medical Oncology, National Center for Tumor Diseases (NCT), Heidelberg University Hospital, Heidelberg, Germany; i Department of Medicine I, Faculty of Medicine and University Hospital Carl Gustav Carus, TUD Dresden University of Technology, Dresden, Germany

**Keywords:** appendectomy, cholecystectomy, minimally invasive surgery, surgical video analysis, vision-language models

## Abstract

**Background:**

The ongoing shortage of medical personnel highlights the urgent need to automate clinical documentation and reduce administrative burden. Large vision-language models (VLMs) offer promising potential for supporting surgical documentation and intraoperative analysis.

**Methods:**

We conducted an observational, comparative performance study of two general-purpose VLMs – GPT-4o (OpenAI) and Gemini-1.5-pro (Google) – from June to September 2024, using 15 cholecystectomy and 15 appendectomy videos (1 fps) from the CholecT45 and LapApp datasets. Tasks included object detection (vessel clips, gauze, retrieval bags, bleeding), surgery type classification, appendicitis grading, and surgical report generation. In-context learning (ICL) was evaluated as an enhancement method. Performance was assessed using descriptive accuracy metrics.

**Results:**

Both models identified vessel clips with 100% accuracy. GPT-4o outperformed Gemini-1.5-pro in retrieval bag (100% vs. 93.3%) and gauze detection (93.3% vs. 60%), while Gemini-1.5-pro showed better results in bleeding detection (93.3% vs. 86.7%). In surgery classification, Gemini-1.5-pro was more accurate for cholecystectomies (93% vs. 80%), with both models achieving 60% accuracy for appendectomies. Appendicitis grading showed limited performance (GPT-4o: 40%, Gemini-1.5-pro: 26.7%). For surgical reports, GPT-4o produced for CCE more complete outputs (CCE: 90.4%, APE: 80.1%), while Gemini-1.5-pro achieved higher correctness overall (CCE: 71.1%, APE: 69.6%). ICL notably improved tool recognition (e.g., in APE step 4, GPT-4o improved from 69.2% to 80%), though its effect on organ removal step recognition was inconsistent.

**Conclusion:**

GPT-4o and Gemini-1.5-pro performed reliably in object detection and procedure classification but showed limitations in grading pathology and accurately describing procedural steps, which could be enhanced through in-context learning. This shows that domain-agnostic VLMs can be applied to surgical video analysis. In the future, VLMs with domain knowledge can be envisioned to enhance the operating room in the form of companions.

## Introduction

Large language models (LLMs) have demonstrated potential in medical applications, particularly in medical writing and documentation^[1]^. LLMs exhibit broad medical knowledge comparable to clinical guidelines and textbooks^[2]^. They have shown remarkable success in passing medical exams like the United States Medical Licensing Examination, providing accurate justifications for their answers^[3,4]^. Building on the success of LLMs, vision languages models (VLMs) have introduced new possibilities by integrating computer vision with LLMs^[5]^. General-purpose VLMs like GPT-4o by OpenAI or Gemini-1.5-pro by Google are available for multiple use cases and have been evaluated in medical image processing^[6-8]^. VLMs could transform medical fields that rely on visual data, such as radiology, pathology, and surgery by assisting in diagnosis or documentation^[9-11]^. However, literature remains inconclusive on whether general-purpose VLMs or specialized models like PathChat and MedPaLM are optimal for medical applications^[12-14]^. Due to broadly available minimally invasive surgery (MIS) and the rising availability of robotic surgery, VLMs hold the potential to play a critical role in the future of surgery by advancing the analysis and processing of intraoperative video data^[15]^. Currently, surgical image analysis relies on machine learning systems^[16,17]^. VLMs could contribute to the goal of advancing surgical image analysis, such as quality improvement or intraoperative decision support, or alleviate documentational burden^[18]^. AI-generated surgery reports of prostatectomies have already demonstrated higher accuracy than those written by surgeons a recent study^[19]^. Medical staff spend up to one-third of their time on managing electronic medical records, highlighting the need for more efficient solutions^[20,21]^. Specifically, the documentation of interventions is a time-consuming and repetitive task which could be augmented by new technology in the future^[22]^. In the present study, we systematically evaluate whether general-purpose VLMs can perform surgical vision tasks of varying complexity, benchmarking GPT-4o against Gemini-1.5-pro. We demonstrate that VLMs can accurately detect surgical objects and identify the type of surgery from APE and CCE videos. We show that general purpose VLMs can generate rudimentary surgical reports, with limitations in accurately recognizing crucial procedural steps. Finally, we show that model performance can be augmented using in-context learning (ICL). All experiments have been conducted in accordance with the TITAN guidelines for the responsible reporting of research which has been augmented with the use of artificial intelligence (Supplementary Digital Content Table 1, available at: http://links.lww.com/JS9/E690).^[23]^

## Methods

### Experimental design

**Figure 1.**
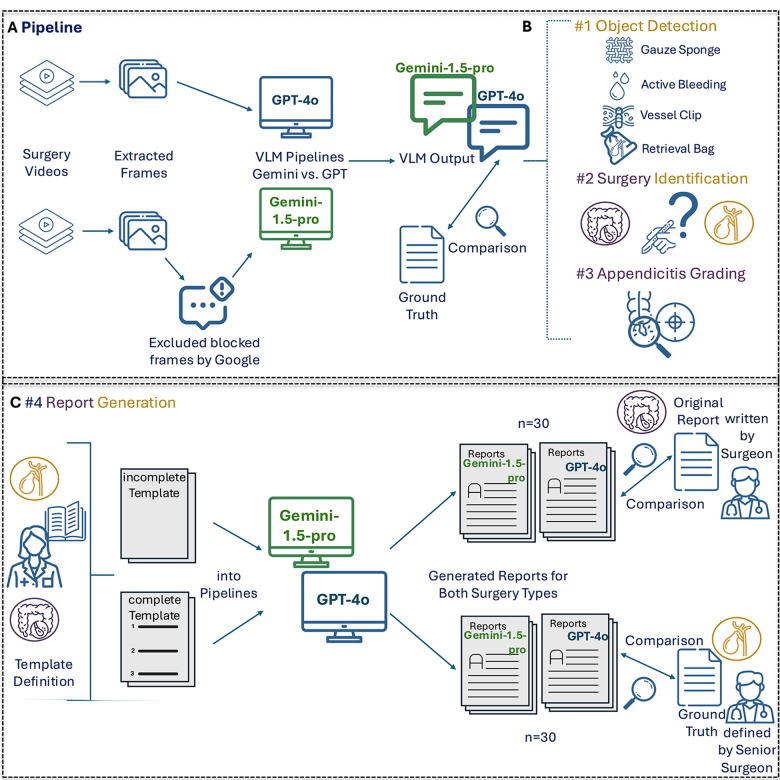
Experimental design. A: Experimental Setup: Fifteen videos from each dataset were randomly selected and processed at a rate of one frame per second. These frames were then integrated into image processing pipelines using GPT-4o and Gemini-1.5-pro, respectively, utilizing their respective Application Programming Interface (API). VLM output was assessed against the ground truth for model perfroance evaluation. B: Summary of the first three tasks: object detection, surgery type identification, and appendicitis grading. All results were then compared to their respective ground truth values. C: Generation of surgical reports. For both surgery types, an incomplete template and a complete template were defined and integrated into the pipelines. The models generated two reports for each video. All reports were compared against their corresponding, pre-defined ground truth. All icons were obtained from https://www.flaticon.com/.

We evaluated two general-purpose VLMs on CCE and APE video analysis through four increasingly complex tasks. In our workflow, the video frames, together with pre-defined system and user prompts, are processed by GPT-4o or Gemini-1.5-pro, respectively, which were accessed via their respective Application Programming Interfaces (APIs) (Fig. 1A). The system prompt directs the VLM to analyze video frames, answer questions, and pinpoint the frame containing the requested information, relying solely on the visual input. The user prompt specifies the respective image analysis task (Supplementary Digital Content Table 2, available at: http://links.lww.com/JS9/E690). We compared GPT-4o by OpenAI (accessed June 18 –14 August 2024) and Gemini-1.5-pro by Google (accessed July 1– September 3 2024). The first task involved the detection of surgical objects (surgical gauze sponge, vessel clips, and retrieval bags) and identification of active bleeding (Fig. 1B). This task was performed on the CCE dataset for benchmarking purposes against established methods^[24-30]^. The second task involved the identification of the type of surgery. This was performed on both datasets (Fig. 1B). The third task involved macroscopic appendicitis grading based on Gomes *et al*^[31]^ (Supplementary Digital Content Table 3, available at: http://links.lww.com/JS9/E690), which was performed on the APE dataset (Fig. 1B). The grading system was incorporated into the user prompt as depicted in Supplementary Digital Content Table 3, available at: http://links.lww.com/JS9/E690. The final task involved the generation of a descriptive surgical report, which was conducted on both datasets (Fig. 1C) without and with in-context learning (ICL)^[32]^.

**HIGHLIGHTS**

- GPT-4o and Gemini-1.5-pro accurately identified surgical objects and classified surgery types from laparoscopic videos, with accuracies up to 100%.
- Gemini-1.5-pro generated more accurate surgical reports, while GPT-4o produced more comprehensive procedural descriptions.
- GPT-4o outperformed Gemini-1.5-pro in appendicitis grading; however, performance for this task remained subpar.
- Vision-language models (VLMs) excelled in surgical object and procedure recognition.
- VLM-assisted surgical reporting benefited from structured guidance.
- Both models struggled most with grading appendicitis severity.

### Image analysis setup

Fifteen videos were randomly selected per dataset. From the APE dataset, video frames were extracted at a rate of one frame per second (fps); the CCE dataset was already available as frames at a rate of 1 fps. All frames were resized to 428 × 240 pixels at 90 dpi in *jpeg* format.

For GPT-4o, the images were first converted to base64 format and then, together with the respective system and user prompts, sent to the OpenAI API for processing. For data efficiency purposes, this was done with every fifteenth frame. Based on the input images and accompanying instructions, the API generated a response. For Gemini-1.5-pro, the model was initiated with the respective system prompt. Every fifteenth image was sent to the Gemini API without further processing (this is required by Gemini). Due to safety mechanisms implemented by Gemini, some frames were flagged for harmful content by the model (Supplementary Digital Content Table 4, available at: http://links.lww.com/JS9/E690). Those frames had to be excluded, which we achieved by iteratively querying the API and discarding flagged frames until only non-harmful images were uploaded to the API together with the user prompt. Image categories that trigger safety mechanisms include “harassment,” “sexually explicit” and “dangerous” content (see Gemini for further information^[33]^). Based on the input images and accompanying instructions, the API generated a response.

### Generation of surgical reports

For the surgical reports, we created templates outlining the key steps for each type of surgery, serving as a guideline for the model (Supplementary Digital Content Tables 5 and 6, available at: http://links.lww.com/JS9/E690). In a first approach, a list of the key steps of the respective surgical procedure was incorporated into the user prompt. In a second approach, that list was extended to include a best practice outline of each step for the VLM to modify according to the respective video content. Each step of the template, along with its detailed outline, was developed based on state-of-the-art procedures for CCEs and APEs^[34,35]^.

Each model generated two reports per video: one independently completing the template and the other modifying a pre-defined standard report. For APEs, we repeated the analysis using ICL to improve accuracy in steps initially performed inaccurately. Images appended for ICL included endoloops, retrieval bags, scissors, vessel clips, and appendix removal methods without a retrieval bag. These images were taken from remaining APE dataset videos not included in the main experiments to prevent data leakage.

### Annotation, evaluation and statistics

For CCE object detection, a trained observer manually reviewed frames to establish the ground truth. These annotations were compared to VLM outputs. For the appendicitis grading, the predefined appendicitis grades were provided by the source hospital for compilation of the LapApp dataset. The VLM-generated grades were then evaluated against the clinical grades on a video level. For the CCE surgical reports, the ground truth was established by a senior surgeon who reviewed all fifteen CCE videos and pre-specified the minimum of information that needed to be included in a report for every step outlined in the best-practice guideline for each video. This approach allowed us to create a ground truth aligned precisely with the template, enabling a direct comparison with the VLM-generated reports. For the APEs, the original reports and the VLM-generated reports were compared against the original video.

**Figure 2.**
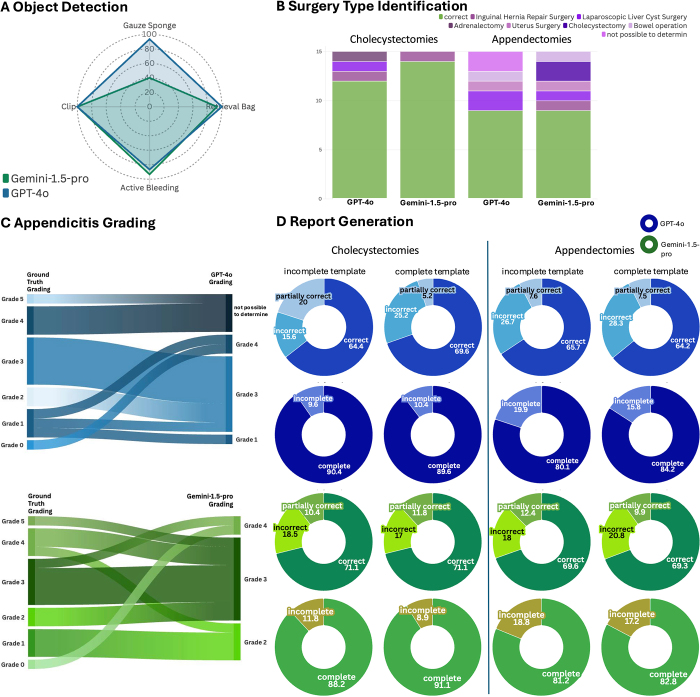
Results. A: Radar plot showing model performance in object detection tasks for GPT-4.0 and Gemini-1.5-pro. B: Stacked bar charts showing Surgery type identification outcomes for GPT-4.0 and Gemini-1.5-pro. C: Sankey plot showing appendicitis grading accuracy for GPT-4.0 and Gemini-1.5-pro. D: Assessment of correctness and completeness in report generation. Pie charts on the left represent results for CCE reports, while those on the right pertain to APE reports. Blue sections correspond to GPT-4.0s, and green sections represent Gemini-1.5-pros results. All graphics were created with flourish https://flourish.studio/.

**Figure 3.**
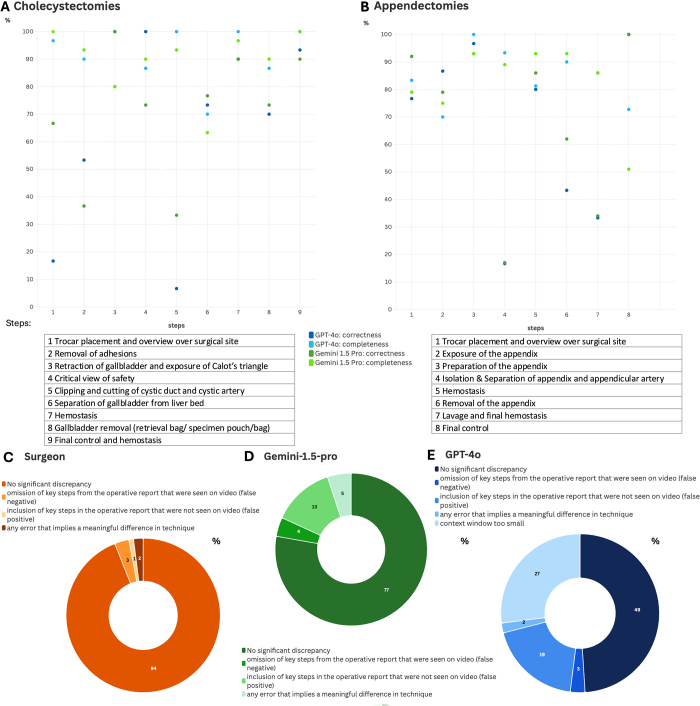
In-depth analysis of surgical report quality. A: Completeness and correctness of surgical reports stratified by surgical step for CCEs. B: Completeness and correctness of surgical reports stratified by surgical step for APEs. C: Donut chart showing quantitative analysis of clinically relevant errors in original APE surgical reports. D: Donut chart showing quantitative analysis of clinically relevant errors in APE surgical reports generated by Gemini-1.5-pro. E: Donut chart showing quantitative analysis of clinically relevant errors in APE surgical reports generated by GPT-4o. All results with complete numbers can be found in Suppl. Tables 6 and 7. All graphics were created with flourish https://flourish.studio/.

To assess the APE and CCE reports, two criteria were applied for each step: completeness (yes vs. no) and correctness (yes vs. partially correct, vs.no). The percentage of correct and complete reports was calculated for each video. The average correctness and completeness were determined separately for both report types (incomplete vs. complete template) to compare overall performance. We compared the overall performance of the reports from each group as well as the correctness and completeness of each individual step across all reports. This allowed for a detailed assessment of both the general report quality and step-specific performance differences (Fig. 2 D, Fig. 3). All generated reports are attached in the supplementary material (Supplementary Digital Content Table 7, available at: http://links.lww.com/JS9/E690).

For an in-depth quality assessment, the AI-generated and the original surgeon-generated reports were reviewed for discrepancies with the video content of APEs, where original reports were available. Discrepancies were classified as clinically significant, defined as key omissions (1a), false inclusions (1b), or other technique-altering errors (1c) or not clinically significant^[2]^ as described before.^[19]^

## Results

### General purpose VLMs accurately identify surgical objects in APE and CCE videos

For the detection of vessel clips, GPT-4o and Gemini-1.5-pro both identified the presence of vessel clips in 100% of cases (1.0 precision (p), 1.0 recall (r), 1.0 F1-score (F1)). GPT-4o correctly detected the retrieval bag in 100% of cases (1.0 p, 1.0 r, 1.0 F1), while Gemini-1.5-pro achieved an accuracy of 93.3% (14 out of 15 cases, 1.0 p, 0.93 r, 0.97 F1). In detecting the use of surgical gauze sponges, GPT-4o was accurate in 93.3% (14 out of 15 cases, 0.5 p, 1.0 r, 0.67 F1), whereas Gemini-1.5-pro reached 60% accuracy (9 out of 15 cases, 14 out of 15 cases, 1 p, 1.0 r, 0.18 F1). For detecting active bleeding, GPT-4o had an accuracy of 86.7% (13 out of 15 cases, 14 out of 15 cases, 0.93 p, 0.93 r, 0.93 F1), while Gemini-1.5-pro performed slightly better at 93.3% (14 out of 15 cases, 0.93 p, 1.0 r, 0.97 F1) (Table 1, Fig. 2A). In conclusion, GPT-4o outperformed Gemini-1.5-pro in detecting gauze sponges, however, both models demonstrated highly accurate object detection performance.

**Table 1:** Accuracy, precision, recall and F1-score performance in object detection task for both models

	Accuracy (number of correctly identified cases/total number of cases)		Precision		Recall		F1-score	
Object detection	GPT-4o	Gemini-1.5-pro	GPT-4o	Gemini-1.5-pro	GPT-4o	Gemini-1.5-pro	GPT-4o	Gemini-1.5-pro
Vessel clip	1.00	1.00	1.00	1.00	1.00	1.00	1.00	1.00
	15/15	15/15						
	(100%)	(100%)						
Retrieval bag	1.00	0.93	1.00	1.00	1.00	0.93	1.00	0.97
	15/15	14/15						
	(100%)	(93%)						
Gauze sponge	0.93	0.40	0.5	0.1	1.00	1.00	0.67	0.18
	14/15	6/15						
	(93%)	(49%)						
Bleeding	0.87	0.93	0.93	0.93	0.93	1.00	0.93	0.97
	13/15	14/15						
	(87%)	(93%)						

All values are presented in decimal form, where 1.00 corresponds to 100%.

**Table 2:** Effects of in-context learning

GPT-4o		Out-of-the-box model	In-context learning
Separation tool	Approach #1	69.23%	80%
	Approach #2	53.34%	66.67%

Way of removal	Approach #1	75%	77.78%
	Approach #2	60%	50%
**Gemini-1.5-pro**			
Separation tool	Approach #1	50%	78.57%
	Approach #2	26.67%	60%

Way of removal	Approach #1	57.14%	75%
	Approach #2	73.34%	73.34%

Effects of ICL on accurately defining the proper procedure for separating the appendix, as well as the methods for its removal from the surgical site. Reports generated with incomplete template (approach #1) and modifying a complete (approach #2).

### General purpose VLMs can identify the type of surgery from MIS video material

GPT-4o identified CCEs correctly in 80% of cases, while Gemini-1.5-pro achieved 93% (Fig. 2B). Both models correctly identified APEs in 60% of cases. GPT-4o failed two cases due to context length limits, and misclassified some videos as other surgeries (Fig. 2B). Hence, general purpose VLMs exhibit the ability to identify a surgical procedure from MIS video material without any prior instruction, however, the success rate in CCEs was higher than in APEs.

### Inaccurate appendicitis grading by VLMs based on the Gomes classification

After pre-specification of the laparoscopic grading system of appendicitis proposed by Gomes *et al*, GPT-4o accurately determined the appendicitis grade in 6 out of 15 cases, achieving a success rate of 40%. Of these, 1 out of 3 Grade 1 cases was correctly identified, all 5 Grade 3 cases were classified accurately, and the remaining cases were misclassified (Fig. 2C). In another 4 out of 15 cases, the context window was exceeded and a grade could not be provided. Gemini-1.5-pro graded 26.7% of the appendicitis correctly (4 out of 15 cases). All correctly identified cases were Grade 3, meaning 4 out of 5 Grade 3 cases were classified accurately, while the other grades were misclassified (Fig. 2C).

To conclude, almost all of the correct gradings were Grade 3 appendicitis, however, neither model was able to reliably determine the correct grade of appendicitis.

### VLMs can generate rudimentary surgical reports, but the identification of crucial steps is subpar

For CCE surgical reports, both models performed similarly. With incomplete templates, GPT-4o achieved 90.4% completeness, slightly exceeding Gemini-1.5-pro’s 88.1%. However, Gemini-1.5-pro had higher correctness (71.1% vs. 64.4%) (Fig. 2D). With a best practice outline, Gemini-1.5-pro showed slightly higher completeness (91.1% vs. GPT-4o’s 89.6%) and outperformed in correctness (71.1% vs. 69.6%) (Fig. 2D). The same analysis for APEs showed Gemini-1.5-pro had higher completeness (81.2% vs. 80.1%) and correctness (69.6% vs. 65.7%) in the first approach. In the second, GPT-4o had 84.2% completeness and 64.2% correctness, while Gemini-1.5-pro had 82.8% completeness but a higher correctness of 69.3% (Fig. 2D). Step-wise analysis revealed that for APEs, “*Isolation and Separation of the Appendix, and Appendicular Artery*” had the lowest performance, with Gemini-1.5-pro at 17.4% correctness and 89.6% completeness, while GPT-4o scored 16.7% and 93.3% (Fig. 3B, Supplementary Digital Content Table 8, available at: http://links.lww.com/JS9/E690). In CCEs, “*Clipping and cutting the cystic duct and cystic artery*” was the most challenging step, with Gemini-1.5-pro achieving 33.3% correctness and 100% completeness, while GPT-4o scored 6.7% and 93.3%, respectively (Fig. 3A, Supplementary Digital Content Table 9, available at: http://links.lww.com/JS9/E690). Comparing surgeon-generated vs. AI-generated APE documentation, an in-depth quality assessment revealed that the surgeon’s operative reports showed 112 instances without significant discrepancies (94%), 4 false negatives (3.4%), 1 false positive (0.8%), and 2 technique-related errors (1.7%) (Fig. 3C). Gemini-1.5-pro generated reports with 91 cases showing no significant discrepancies (77%), 4 false negatives (3.6%), 15 false positives (13.5%), and 6 technique-related errors (5.0%) (Fig. 3B). GPT-4o reports included 59 cases without significant discrepancies (49.2%), 3 false negatives (2.5%), 23 false positives (19.2%), and 3 technique-related errors (2.5%). Additionally, 32 steps (26.7%) were marked as incomplete due to a limited context window (Fig. 3C). Overall, the surgeon reports showed the highest accuracy with the fewest discrepancies, followed by Gemini-1.5-pro, which outperformed GPT-4o. To conclude, both models struggled with organ separation, highlighting it as a key area for improvement. Providing a best practice outline enhanced both completeness and correctness, with Gemini-1.5-pro excelling in correctness and GPT-4o maintaining slightly higher completeness.

### In-context learning improves model accuracy

Due to the limited accuracy in the identification of the removal mechanism of the target organ as described above, we repeated the analysis with additional information provided through ICL for step four (“*Isolation & Separation of appendix and appendicular artery”)* in the example of APEs. ICL significantly improved the detection of the correct separation tool, with accuracy in approach #1 (incomplete template) increasing from 69.2% to 80% and approach #2 (complete template) rising from 53.3% to 66.7% for the respective substep. In contrast, the impact of ICL on detecting the correct removal method was inconsistent. In approach #1, the performance showed a slight improvement, increasing from 75% to 77.8%, while in approach #2 it declined from 60% to 50%, indicating variability in performance across different tasks (Table 2). Overall, ICL increased model performance.

## Discussion

In the present study, we evaluated VLMs for surgical video analysis in CCEs and APEs. Without further training, general-purpose VLMs accurately identified objects and surgery types but struggled to grade appendicitis using the Gomes classification. Surgical report generation showed comparable performance across procedures, with most steps achieving around 70% correctness and completeness. Identification of crucial intraoperative steps, like resection methods in APEs, was initially low but was improved with ICL.

There are already comparable studies on deep learning (DL)-assisted surgical instrument classification, anatomy classifications, surgical phase recognition, and critical view of safety assess-ments^[24-30]^. To include an assessment of VLMs for a range of classification tasks, we included object detection, procedure type identification and appendicitis grading in our study. We found that both VLMs performed well in simple object detection but struggled in more clinically complex classification tasks. For example, Wu *et al* report a CV-based model achieving around 95% accuracy in disease severity detection for cholecystitis^[36]^, whereas in our results, appendicitis severity grading accuracy was clinically non-viable. Moreover, Nwoye *et al* assessed DL for the identification of instrument-action-target triplets in CCE videos, where the average precision was around 80 for instrument detection, 53 for action recognition, and 47 for target anatomical structure^[37]^. While the majority of operative steps in our analyses have been correctly reported by the VLMs, the lowest accuracies occurred in critical steps outlining the organ removal methods, which involves instrument detection and action recognition simultaneously and leaves room for improvement. In line with comparable studies in pathology, ICL enhanced the performance, which can conceivably refine VLMs for surgical vision tasks in future applications^[38]^. Unlike convolutional neural networks (CNNs), which provide the architecture behind DL and are tailored on task-specific datasets, the applied VLMs are general-purpose models. This allows them to perform a variety of vision and language tasks with zero-shot or few-shot capabilities without requiring additional task-specific training. The main strength of VLMs becomes apparent in generative tasks, like surgical report generation. Khanna *et al* recently showed that in surgical operative reports in robotic-assisted radical prostatectomy a computer-vision (CV) algorithm achieved higher accuracy than those written by surgeons^[19]^. In our work, surgeon-generated APE reports showed a higher accuracy than AI-generated reports, however, our AI-generated reports also exceeded the accuracy of the surgeon-generated reports in the study by Khanna *et al* (77% vs. 72.8%).^[19]^ Conclusively, while our results suggest an inferiority of VLMs in classification tasks, we highlight their potential for free-text surgical documentation, where CNNs fall short. Additionally, VLMs provide the possibility for interactions, explanations and justifications. VLMs can potentially be employed in the form of a companion in the operating room, while leaving the ultimate autonomy to the surgeon. Moreover, it would be imaginable to generate patients-friendly surgical reports or to adapt the VLM output to the state of training of the performing surgeon. Focusing on common procedures, we lay the groundwork for future research by evaluating the performance of VLM-generated surgery reports, which require a complex understanding of the procedure, and exploring ICL for refinement of this technology. After observing the improvement in accuracy following the application of ICL, it becomes evident that this technology can be used to enhance performance and should be explored in future studies. With the advent of autonomous agents for task orchestration in the context of VLMs, we envision VLMs to augment surgical documentation in the future, while sub-tasks which require fine-grained assessments will be completed by CV-based models or enhanced with ICL as needed^[39]^.

To our knowledge, this is among the first studies showcasing automated video-based AI surgical documentation with VLMs. The integration of AI into surgical practices offers promising advancements. However, ensuring physician oversight remains crucial for patients and healthcare professionals^[40,41]^.

## Limitations

This study has several limitations. First, we included only two types of surgeries, limiting the representativeness of our results. The availability of publicly accessible abdominal surgery datasets is scarce, which may have contributed to the lower accuracy in identifying APEs compared to CCEs. Potential data leakage could explain this, as publicly available CCE videos may have been included in the VLM training datasets. Moreover, it is preferable to use local models to process sensitive medical data, as proposed in comparable studies^[42,43]^. However, data-heavy applications like surgical video analysis warrant models with a large context window, and the models used in the present study are among the largest available. Nonetheless, due to model constraints, we had to reduce the frame rate to 1 fps, and despite using the largest available models, GPT-4o’s context window was exceeded in longer videos^[44]^. Moreover, some videos were blocked by Gemini due to their content policies, necessitating even more frames to be excluded, which could of course affect performance^[45]^. However, upon reviewing the number and position of blocked frames, they were typically isolated single frames spaced fairly far apart. Despite this circumstance, Gemini-1.5-pro outperformed GPT-4o in many tasks, which could also be attributed to the context window. With current models, there always has to be a trade-off between the level of detail in the prompt and the size of the video, requiring the prompts to be as short as possible. Additionally, reproducibility has long been a significant challenge in science and remains a critical issue in the field^[46]^. It has been demonstrated that the same prompt provided to the same LLM does not always produce identical outputs. This unpredictability creates significant challenges for replicating results and validating findings, highlighting an ongoing problem in ensuring the reliability of experiments and conclusions in VLM research^[47]^.

## Conclusion

This study provides a comprehensive evaluation of general-purpose VLMs for surgical video analysis, demonstrating their ability to accurately detect surgical instruments, identify types of procedures, and generate rudimentary surgical reports. While both GPT-4o and Gemini-1.5-pro showed promising results, especially in object detection and procedural classification, they struggled with more complex tasks like appendicitis grading and detailed documentation of critical intraoperative steps. However, performance improved with the integration of ICL, highlighting the potential of such techniques to enhance accuracy in surgical applications. The field of VLMs is advancing rapidly, with emerging models tailored to surgical contexts. Future work should compare general-purpose and domain-specific VLMs and expand beyond APEs and CCEs to cover a broader range of minimally invasive surgeries.

## Data Availability

The CholecT45 dataset of the CCEs from CAMMA research group is available at https://github.com/CAMMA-public/cholect45. The APE videos are a single-institution subset of the multi institutional LapApp dataset, which will be made publicly available in a separate publication. Use of the APE dataset was approved by the contributing institution. All data were used under a data-sharing agreement ensuring anonymization and ethical compliance. Our code is publicly available at https://github.com/hm828721/SurgicalVideoAnalysis under an MIT license. All video data were fully de-identified prior to use, including manual deletion of any frames containing potentially identifiable features. Data usage complied with all applicable privacy regulations, including GDPR.

## Research registration unique identifying number (UIN)

Not applicable. The present study did not recruit subjects.

## References

[1] Meng X Yan X Zhang K. The application of large language models in medicine: a scoping review. iScience 2024;27:109713. 38746668 10.1016/j.isci.2024.109713 PMC11091685

[2] Thirunavukarasu AJ Ting DSJ Elangovan K Gutierrez L Tan TF Ting DSW. Large language models in medicine. Nat Med 2023;29:1930–40. 37460753 10.1038/s41591-023-02448-8

[3] Kung TH Cheatham M Medenilla A. Performance of ChatGPT on USMLE: potential for AI-assisted medical education using large language models. PLOS Digit Health 2023;2:e0000198. 36812645 10.1371/journal.pdig.0000198 PMC9931230

[4] Gilson A Safranek CW Huang T. Correction: how does ChatGPT perform on the United States medical licensing examination (USMLE)? The implications of large language models for medical education and knowledge assessment. JMIR Med Educ 2024;10:e57594. 38412478 10.2196/57594 PMC10933712

[5] Zhang J Huang J Jin S Lu S. Vision-language models for vision tasks: a survey. IEEE Trans Pattern Anal Mach Intell 2024;46:5625–44. 38408000 10.1109/TPAMI.2024.3369699

[6] Van MH Verma P Wu X On large visual language models for medical imaging analysis: an empirical study. In: 2024 IEEE/ACM Conference on Connected Health: Applications, Systems and Engineering Technologies (CHASE). IEEE; 2024. p. 172–76.

[7] OpenAI AJ Adler S Agarwal S. GPT-4 Technical Report [Internet]. arXiv [cs.CL]. 2023. http://arxiv.org/abs/2303.08774.

[8] Team G Anil R Borgeaud S. Gemini: a family of highly capable multimodal models [Internet]. arXiv [cs.CL]. 2023. http://arxiv.org/abs/2312.11805.

[9] Liu C Jin Y Guan Z. Visual–language foundation models in medicine. Visual Comput [Internet]. 2024. 10.1007/s00371-024-03579-w

[10] Onitilo AA Shour AR Puthoff DS Tanimu Y Joseph A Sheehan MT. Evaluating the adoption of voice recognition technology for real-time dictation in a rural healthcare system: a retrospective analysis of dragon medical one. PLoS One 2023;18:e0272545. 36952436 10.1371/journal.pone.0272545 PMC10035815

[11] Buckley T Diao JA Rodman A Manrai AK Accuracy of a vision-language model on challenging medical cases [Internet]. arXiv [cs.CV]. 2023. http://arxiv.org/abs/2311.05591.

[12] Wang Z Wang H Danek B. A perspective for adapting generalist AI to specialized medical AI applications and their challenges [Internet]. arXiv [cs.CL]. 2024. http://arxiv.org/abs/2411.00024. 10.1038/s41746-025-01789-7 PMC12254199 40646157

[13] Nori H Lee YT Zhang S. Can generalist foundation models outcompete special-purpose tuning? Case study in medicine [Internet]. arXiv [cs.CL]. 2023. http://arxiv.org/abs/2311.16452.

[14] Wang J Ning H Peng Y. A survey on large language models from general purpose to medical applications: datasets, methodologies, and evaluations [Internet]. arXiv [cs.CL]. 2024. http://arxiv.org/abs/2406.10303.

[15] Sheetz KH Claflin J Dimick JB. Trends in the adoption of robotic surgery for common surgical procedures. JAMA Network Open 2020;3:e1918911. 31922557 10.1001/jamanetworkopen.2019.18911 PMC6991252

[16] Kiyasseh D Ma R Haque TF. A vision transformer for decoding surgeon activity from surgical videos. Nat Biomed Eng 2023;7:780–96. 36997732 10.1038/s41551-023-01010-8 PMC10307635

[17] Nwoye CI Yu T Gonzalez C. Rendezvous: attention mechanisms for the recognition of surgical action triplets in endoscopic videos. Med Image Anal 2022;78:102433. 35398658 10.1016/j.media.2022.102433

[18] Mascagni P Alapatt D Sestini L. Computer vision in surgery: from potential to clinical value. NPJ Digit Med 2022;5:163. 36307544 10.1038/s41746-022-00707-5 PMC9616906

[19] Khanna A Wolf T Frank I. Enhancing accuracy of operative reports with automated artificial intelligence analysis of surgical video. J Am Coll Surg 2025;240:739–46. 39918224 10.1097/XCS.0000000000001352

[20] Tipping MD Forth VE O’Leary KJ. Where did the day go?–a time-motion study of hospitalists. J Hosp Med 2010;5:323–28. 20803669 10.1002/jhm.790

[21] Sinsky C Tutty M Colligan L. Allocation of physician time in ambulatory practice. Ann Intern Med 2017;166:683–84. 10.7326/L17-0073 28460382

[22] Ray PP. Large language models in laparoscopic surgery: a transformative opportunity. Laparosc Endosc Robot Surg 2024;7:174–80.

[23] Agha RA Mathew G Rashid R. Transparency in the Reporting of Artificial Intelligence – The TITANGuideline. Premier Journal of Science 2025;10:100082.

[24] Kiyasseh D Ma R Haque TF. Quantification of robotic surgeries with vision-based deep learning[Internet]. arXiv [cs.RO]. 2022. http://arxiv.org/abs/2205.03028.

[25] Schmidgall S Cho J Zakka C Hiesinger W GP-VLS: a general-purpose vision language model for surgery[Internet]. arXiv [cs.CV]. 2024. http://arxiv.org/abs/2407.19305.

[26] Wang G Bai L Nah WJ. Surgical-LVLM: learning to adapt large vision-language model for grounded visual question answering in robotic surgery [Internet]. arXiv [cs.CV]. 2024. http://arxiv.org/abs/2405.10948.

[27] Li J Quaranto BR Xu C. Recognize any surgical object: unleashing the power of weakly-supervised data [Internet]. arXiv [cs.CV]. 2025. http://arxiv.org/abs/2501.15326.

[28] Liu Y Boels M Garcia-Peraza-Herrera LC. LoViT: long video transformer for surgical phase recognition. Med Image Anal 2025;99:103366. 39418831 10.1016/j.media.2024.103366 PMC11876726

[29] Cerón JCÁ Ruiz GO Chang L Ali S. Real-time instance segmentation of surgical instruments using attention and multi-scale feature fusion. Med Image Anal 2022;81:102569. 35985195 10.1016/j.media.2022.102569

[30] Kawamura M Endo Y Fujinaga A. Development of an artificial intelligence system for real-time intraoperative assessment of the critical view of safety in laparoscopic cholecystectomy. Surg Endosc 2023;37:8755–63. 37567981 10.1007/s00464-023-10328-y

[31] Gomes CA Sartelli M Di Saverio S. Acute appendicitis: proposal of a new comprehensive grading system based on clinical, imaging and laparoscopic findings. World J Emerg Surg 2015;10:60. 26640515 10.1186/s13017-015-0053-2 PMC4669630

[32] Doveh S Perek S Mirza MJ. Towards multimodal in-context learning for vision & language models [Internet]. arXiv [cs.CV]. 2024. http://arxiv.org/abs/2403.12736.

[33] Google AI for Developers. [Internet]. Accessed 29 Jan 2025. Generating content. https://ai.google.dev/api/generate-content?hl=de#v1beta.HarmCategory.

[34] Hassler KR Collins JT Philip K Jones MW. Laparoscopic cholecystectomy. In: StatPearls. [Internet]. StatPearls Publishing; 2023.

[35] Gorter RR Heij HA Eker HH Kazemier G. Laparoscopic appendectomy: state of the art. Tailored approach to the application of laparoscopic appendectomy? Best Pract Res Clin Gastroenterol 2014;28:211–24. 24485267 10.1016/j.bpg.2013.11.016

[36] Wu S Chen Z Liu R. SurgSmart: an artificial intelligent system for quality control in laparoscopic cholecystectomy: an observational study. Int J Surg 2023;109:1105–14. 37039533 10.1097/JS9.0000000000000329 PMC10389595

[37] Nwoye CI Alapatt D Yu T. CholecTriplet2021: a benchmark challenge for surgical action triplet recognition. Med Image Anal 2023;86:102803. 37004378 10.1016/j.media.2023.102803

[38] Ferber D Wölflein G Wiest IC. In-context learning enables multimodal large language models to classify cancer pathology images. Nat Commun 2024;15:10104. 39572531 10.1038/s41467-024-51465-9 PMC11582649

[39] Ferber D El Nahhas OSM Wölflein G. Development and validation of an autonomous artificial intelligence agent for clinical decision-making in oncology. Nat Cancer 2025. 10.1038/s43018-025-00991-6 PMC12380607 40481323

[40] Parry MW Markowitz JS Nordberg CM Patel A Bronson WH DelSole EM. Patient perspectives on artificial intelligence in healthcare decision making: a multi-center comparative study. Indian J Orthop 2023;57:653–65. 37122674 10.1007/s43465-023-00845-2 PMC9979110

[41] Layard Horsfall H Palmisciano P Khan DZ. Attitudes of the surgical team toward artificial intelligence in neurosurgery: international 2-stage cross-sectional survey. World Neurosurg 2021;146:e724–30. 33248306 10.1016/j.wneu.2020.10.171 PMC7910281

[42] Nievas M Basu A Wang Y Singh H. Distilling large language models for matching patients to clinical trials. J Am Med Inform Assoc 2024;31:1953–63. 38641416 10.1093/jamia/ocae073 PMC11339497

[43] Wiest IC Ferber D Zhu J. Privacy-preserving large language models for structured medical information retrieval. NPJ Digit Med 2024;7:257. 39304709 10.1038/s41746-024-01233-2 PMC11415382

[44] OpenAI Platform. [Internet]. Accessed 23 Jan 2025. https://platform.openai.com/docs/overview.

[45] Gemini. [Internet]. Accessed 17 Jan 2025. Policy guidelines for the Gemini app. https://gemini.google/policy-guidelines/.

[46] Belz A Agarwal S Shimorina A Reiter E. A systematic review of reproducibility research in natural language processing. 2021. 10.48550/arXiv.2103.07929

[47] Ouyang S Zhang JM Harman M Wang M. An empirical study of the non-determinism of ChatGPT in code generation. 2023. 10.48550/arXiv.2308.02828

